# Investigation of the Application of Reduced Graphene Oxide–SPION Quantum Dots for Magnetic Hyperthermia

**DOI:** 10.3390/nano14191547

**Published:** 2024-09-25

**Authors:** Haneen Omar, Yara Ahmed Alkurdi, Arshia Fathima, Edreese H. Alsharaeh

**Affiliations:** College of Science and General Studies, Alfaisal University, P.O. Box 50927, Riyadh 11533, Saudi Arabia; homar@alfaisal.edu (H.O.);

**Keywords:** hyperthermia, graphene oxide, SPIONs, quantum dots, cancer treatment, magnetic hyperthermia

## Abstract

Integrating hyperthermia with conventional cancer therapies shows promise in improving treatment efficacy while mitigating their side effects. Nanotechnology-based hyperthermia, particularly using superparamagnetic iron oxide nanoparticles (SPIONs), offers a simplified solution for cancer treatment. In this study, we developed composites of SPION quantum dots (Fe_3_O_4_) with reduced graphene oxide (Fe_3_O_4_/RGO) using the coprecipitation method and investigated their potential application in magnetic hyperthermia. The size of Fe_3_O_4_ nanoparticles was controlled within the quantum dot range (≤10 nm) by varying the synthesis parameters, including reaction time as well as the concentration of ammonia and graphene oxide, where their biocompatibility was further improved with the inclusion of polyethylene glycol (PEG). These nanocomposites exhibited low cytotoxic effects on healthy cells (CHO-K1) over an incubation period of 24 h, though the inclusion of PEG enhanced their biocompatibility for longer incubation periods over 48 h. The Fe_3_O_4_/RGO composites dispersed in acidic pH buffer (pH 4.66) exhibited considerable heating effects, with the solution temperature increasing by ~10 °C within 5 min of exposure to pulsed magnetic fields, as compared to their dispersions in phosphate buffer and aqueous dimethylsulfoxide solutions. These results demonstrated the feasibility of using quantum dot Fe_3_O_4_/RGO composites for magnetic hyperthermia-based therapy to treat cancer, with further studies required to systematically optimize their magnetic properties and evaluate their efficacy for in vitro and in vivo applications.

## 1. Introduction

Uncontrolled cell proliferation and the potential for metastasis make cancer an extremely complex disease that presents many difficulties in terms of management and therapy. Conventional cancer therapies, such as radiation and chemotherapy, can have poor effectiveness and serious adverse side effects [1,2,3,4,5].

A synergistic combination of anticancer therapies, such as hyperthermia therapy for ablation of cancer cells and targeted drug delivery, could possibly mitigate the adverse effects of conventional treatment while simultaneously improving the effectiveness of localized cancer treatment [4,5,6,7]. Magnetic hyperthermia using nanoparticles is a prospective treatment for targeted ablation of cancer cells, where the localized heating of the tumor cells within narrow temperature ranges (43–45 °C) would cause apoptosis or necrosis of the tumor cells [6].

Magnetic hyperthermia is achieved by using iron or ferrite nanoparticles that are made to accumulate selectively in the tumor and then exposed to a magnetic field with subsequent heat generated due to magnetic hysteresis and relaxation losses of the nanoparticles [8]. The effectiveness of magnetic hyperthermia as an anticancer therapy can be further maximized when combined with other treatment methods including targeted drug delivery and radiation [9,10,11,12].

Biocompatible superparamagnetic iron oxide nanoparticles (SPIONs), having a particle size ranging from 10 to 200 nm [13,14], have been widely employed for magnetic hyperthermia applications. The size, morphology and functionalization of SPIONs have significantly influenced their magnetic properties and, consequently, their heating capacities for hyperthermia therapy [14]. Although the critical size for SPIONs to achieve superparamagnetism has been shown to be influenced by their morphology [14], SPIONs with particle sizes of less than 20 nm, particularly in the quantum dot range (<10 nm), suffer from the issues of agglomeration and colloidal instability in aqueous dispersions over time, leading to reduced performance for application in magnetic hyperthermia [13].

The development of SPION-based nanocomposites with graphene oxide and its derivatives, such as reduced graphene oxide (RGO), has addressed these challenges while enhancing the thermal conductivity of nanocomposites for hyperthermia applications, as reviewed previously [15,16]. The inclusion of biocompatible surface coatings on SPIONs [13], such as polyethylene glycol (PEG), has also been shown to overcome the challenges of agglomeration and cytotoxicity for practical applications.

This study builds upon our previous research [17], with a particular focus on tuning the particle size of SPION quantum dots and their composites with reduced graphene oxide (RGO) for application in magnetic hyperthermia at lower dosages to minimize potential cytotoxic effects. This study advances the applications of Fe_3_O_4_ quantum dots in magnetic hyperthermia and its possible synergistic applications with anticancer therapies, such as targeted drug delivery, for effective noninvasive cancer treatment with minimal side effects.

## 2. Materials and Methods

### 2.1. Materials

Iron (III) chloride (FeCl_3_), polyethylene glycol (PEG) (average molecular weight 200 g/mol) and phosphate-buffered saline (PBS) were purchased from Sigma-Aldrich, St. Louis, MO, USA. Ferrous sulfate heptahydrate (FeSO_4_), ammonia (NH_3_) solution (30%) and dimethyl sulfoxide (DMSO) were purchased from Loba Chemie, Mumbai, India. Graphite powder and acidic pH buffer (acetic acid/sodium acetate; pH = 4.66) were purchased from Merck, Darmstadt, Germany. Sulfuric acid (H_2_SO_4_), hydrochloric acid (HCl) and potassium permanganate (KMNO_4_) were purchased from Scharlau, Sentmenat, Spain. Sodium nitrate (NaNO_3_) was purchased from Panreac, Castellar del Vallès, Spain, and hydrogen peroxide (H_2_O_2_) solution (30%) was purchased from VWR Chemicals, Radnor, PA, USA.

### 2.2. Synthesis of Fe_3_O_4_ Quantum Dots

The Fe_3_O_4_ QDs were synthesized via a coprecipitation method [18], with the influence of varied synthesis parameters on the size of the QDs being investigated here. Briefly, FeCl_3_ (81.8 mg) was mixed with FeSO_4_ (137.5 mg) in a beaker containing 50 mL of deionized water via magnetic stirring for 10 min. A specified amount of NH_3_ solution (6, 8 or 10 mL) was then added to the mixture dropwise while stirring for 15 min. The obtained mixture was then heated until 80 °C, with the temperature stably maintained for given reaction times (60, 75 or 90 min). The Fe_3_O_4_ precipitates were then collected from the mixture using a centrifuge (3500 rpm for 6 min), which was followed by centrifugal washing with deionized water and ethanol to remove excess reactants. The obtained samples were finally dried at 80 °C in the oven overnight.

### 2.3. Synthesis of Graphene Oxide (GO)

The modified Hummer method was used to synthesize GO [19,20]. Briefly, 115 mL of H_2_SO_4_ at 0 °C was mixed with 2 g of graphite for 25 min at a stirring speed of 400 rpm in a conical flask placed in an ice bath atop a magnetic stirrer followed by the addition of NaNO_3_ (2.5 g). After 20 min of stirring, KMnO_4_ (20 g) was gradually added to the mixture while maintaining the mixture temperature below 15 °C. The mixture was allowed to mix for 20 min followed by heating to 35 °C for 2 h under vigorous stirring in order to oxidize the graphite. Then, deionized water (230 mL) was added to the mixture gradually under vigorous stirring while keeping the solution temperature below 50 °C with the help of an ice bath. The obtained GO solution was allowed to mix for another 20 min followed by termination of the reactions with the addition of H_2_O_2_ (20 mL). The 10% HCl solution (100 mL) was then added to the mixture to allow removal of unreacted oxidants. This step was followed by centrifugation to obtain GO solids, which were then washed with hot deionized water until their pH was almost neutral. The GO mixture was sonicated for 10 min in a bath sonicator before drying at 60 °C in the oven overnight.

### 2.4. Synthesis of Fe_3_O_4_ /RGO Nanocomposites

The Fe_3_O_4_/RGO nanocomposites were obtained by introducing GO solution to the reaction mixture used to synthesize Fe_3_O_4_ QD. Briefly, varied amounts of aqueous GO solution (2 mg/mL) were added to the mixture of FeCl_3_ and FeSO_4_ in 50 mL deionized water prior to adding the NH_3_ solution as described previously (Section 2.2). The amounts of GO solution (25 µL, 253 µL or 510 µL) were added according to their respective weight percentage (0.1%, 1% or 2%) in the 50 mL reaction mixture. The mixture was then heated at 80 °C for 75 min. The composites were obtained after centrifuging the mixture that was washed with deionized water and ethanol. The obtained solids were then dried at 80 °C in the oven. For coating the nanocomposites with PEG, the above procedure was followed, where 4 mL PEG was added after NH_3_, with the composites being synthesized at a reaction time of 75 min.

### 2.5. Characterization of Nanocomposites

The synthesized nanomaterials were characterized using X-ray diffraction (XRD) (Rigaku Miniflex 600, Tokyo, Japan), UV–Visible spectroscopy (Perkin Elmer Inc., Shelton, CT, USA) and high-resolution transmission electron microscopy (HR-TEM) (JEM-2100F, JEOL, Tokyo, Japan) [17]. A copper grid was used for the HR-TEM sample preparation. The average particle sizes were estimated from the peaks obtained in the X-ray diffraction using the Scherrer equation.

### 2.6. Magnetic Hyperthermia

The potential application of synthesized Fe_3_O_4_/RGO nanocomposites for magnetic hyperthermia was investigated in different media suspensions, including PBS, aqueous DMSO (50%) and acidic pH buffer (pH 4.66), to evaluate their heating efficacy in simulated cell environments using the EasyHeat induction heating setup (Ambrell, Rochester, NY, USA) [17]. The frequency for the generation of an alternating magnetic field was presented by the manufacturer at a constant value of 289 kHz, with the strength of the magnetic field being varied by the current flowing through the coils. The dispersion of nanocomposite in a given medium (concentration of 1.6 mg/mL) was prepared in a glass tube by sonicating the dispersion for 10 min in a bath sonicator. The tube was then placed in the magnetic induction coil (8 turns, 3.7 cm diameter), with a metal clamp being used to hold it in place at its center. The metal clamp was insulated with plastic to avoid any interferences (heating effects) from the clamp itself. The magnetic field was generated at a constant current of 150 A with the dispersions exposed to the field in pulses, where the field was switched on for 5 min followed by an off period for 1 min, with the total period of application not exceeding 35 min. The solution temperatures were immediately measured after exposure to the field and after switching it off. The obtained heating curves were fitted with a polynomial model.

### 2.7. Evaluation of Cytotoxicity Using In Vitro Studies

The cytotoxicity of these nanocomposites was evaluated using in vitro studies on healthy Chinese Hamster Ovary cells (CHO-K1), which were obtained from the American Type Culture Collection (ATCC-CCL-61, Manassas, VA, USA). These cells were cultured in Dulbecco’s modified Eagle’s medium (DMEM) containing 10% fetal bovine serum (FBS) and 1% antibiotics (penicillin–streptomycin) at 37 °C in a humidified atmosphere containing 5% CO_2_. The cells were seeded in a 96-well plate with 10 × 10^3^ cells/well and allowed to reach high confluency (~70%). The culture medium was then replaced with media dispersions containing the nanomaterials, where the cells were incubated with Fe_3_O_4_-based nanocomposites (200 and 320 µg/mL of opti-DMEM) at 37 °C for a period of 24 h and 48 h. The healthy cells that were not exposed to the nanomaterials were considered as untreated control (UTC)/negative control, while cells treated with lysis solution (Triton, Houston, TX, USA) were considered as positive control.

The cytotoxic effects of the nanomaterials were evaluated using an alamarBlue assay according to the manufacturer’s protocol. After 1–2 d of incubation with the nanomaterial-based dispersions, the cell culture medium was replaced with fresh media containing 10% alamarBlue reagent and incubated for another 3–4 h at 5% CO_2_ and 37 °C. Fluorescence spectra were then recorded at λ_ex_ of 570 and λ_em_ of 600 using BioTek Cytation 5™ multimode Microplate Reader-Gen5™ software (https://www.agilent.com, Agilent Technologies, Santa Clara, CA, USA).

## 3. Results and Discussion

### 3.1. Characterization of Fe_3_O_4_/RGO Composites

**Figure 1:**
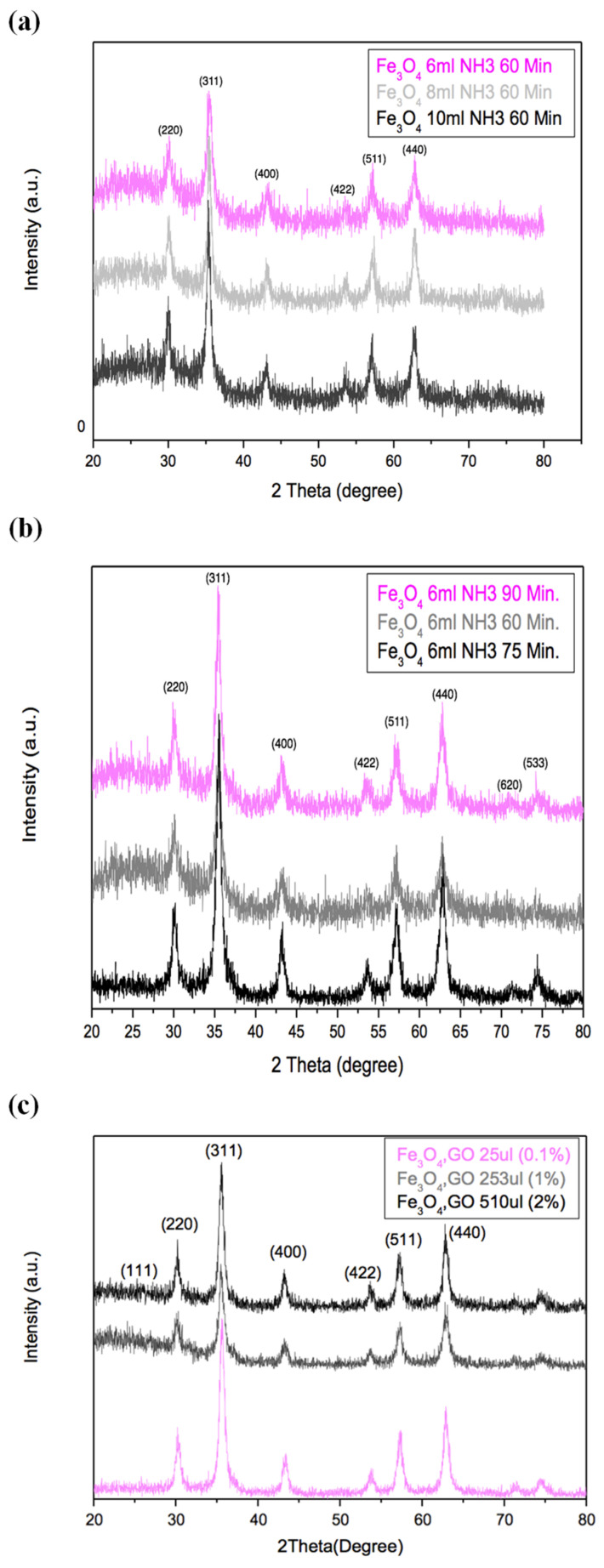
XRD for Fe_3_O_4_ quantum dots synthesized using (**a**) different amounts of ammonia and (**b**) different reaction times, and its composites with RGO obtained by (**c**) varying the amounts of GO solution used during their synthesis.

The XRD peaks observed in the XRD spectra (Figure 1) for synthesized Fe_3_O_4_ in their nanocomposites with RGO aligned with the dominant peaks for magnetite (ICSD 01-087-2334) [17]. The sharp peaks indicated that highly crystalline Fe_3_O_4_ nanoparticles were developed with crystallite sizes ranging between 9 and 14 nm (Table 1), where particle sizes were influenced by the variations in synthesis parameters. Considering the similar sizes of nanoparticles obtained with varied amounts of NH_3_ (Figure 1a), the nanocomposites synthesized with the lowest amounts of the reagent (6 mL NH_3_) were selected for further optimization of the synthesis parameters.

**Figure 2:**
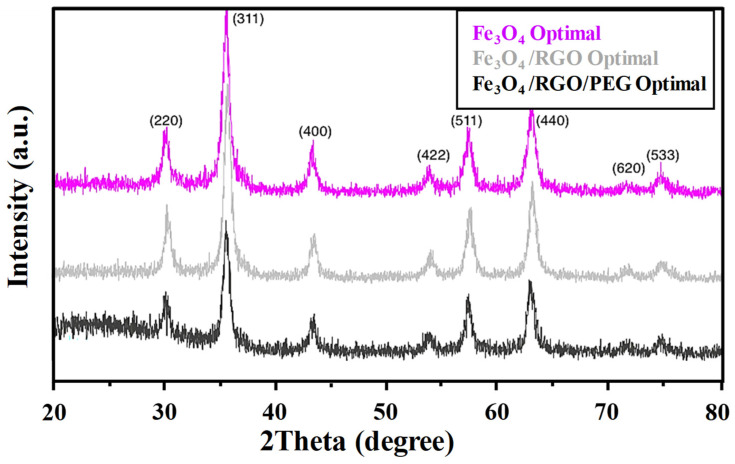
XRD of Fe_3_O_4_/RGO nanocomposites with the inclusion of PEG synthesized at optimal conditions (6 mL NH_3_, 25 µL GO solution and 75 min reaction time).

On the other hand, it was noted that prolonging the reaction time for synthesizing Fe_3_O_4_ nanoparticles to 90 min at 80 °C (Figure 1b) led to solvent evaporation, which made it difficult to maintain stable reaction conditions for collecting Fe_3_O_4_ precipitates with higher purity. Conversely, the samples synthesized within a reaction time of 75 min exhibited the highest crystallinity with minimal impurities. Therefore, an optimal reaction time of 75 min was chosen for the further synthesis of Fe_3_O_4_/RGO nanocomposites with/without the inclusion of PEG, which was anticipated to reduce the particle sizes while enhancing their biocompatibility. The XRD analysis of Fe_3_O_4_/RGO nanocomposites (Figure 1c) revealed similar particle sizes for Fe_3_O_4_ irrespective of the amounts of GO solution introduced during their synthesis. Thus, the nanocomposite with 0.1% GO was selected for further synthesis and analysis in order to minimize potential cytotoxic effects anticipated from the presence of RGO [21]. The inclusion of PEG in the Fe_3_O_4_/RGO nanocomposites under these optimized reaction conditions did not affect the crystalline structure of Fe_3_O_4_/RGO as shown in Figure 2.

**Figure 3:**
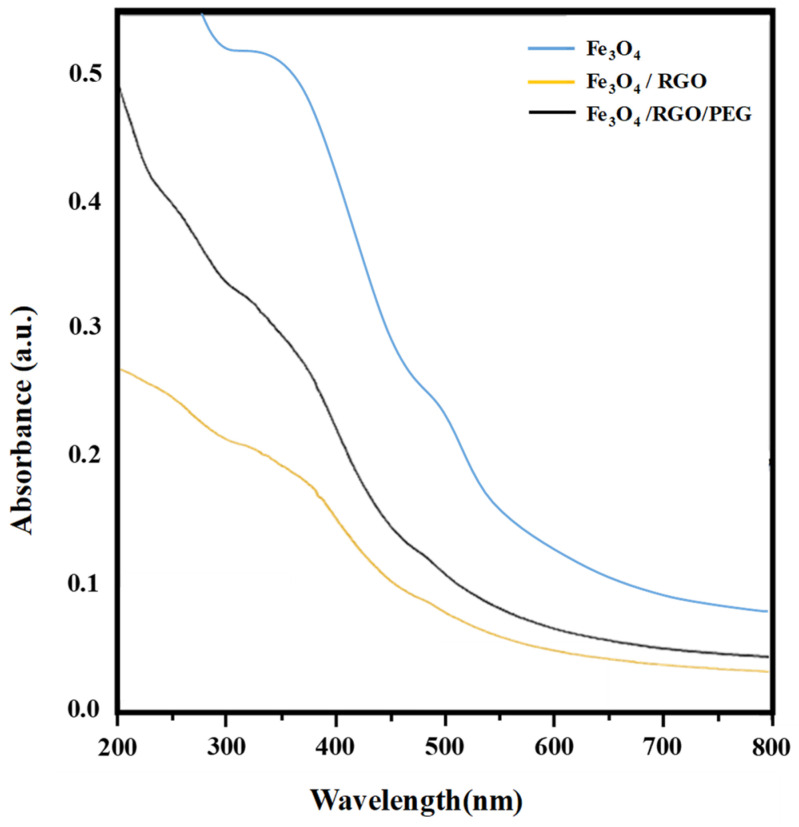
UV-Vis spectra of optimized Fe_3_O_4_, Fe_3_O_4_/RGO and Fe_3_O_4_/RGO/PEG.

**Figure 4:**
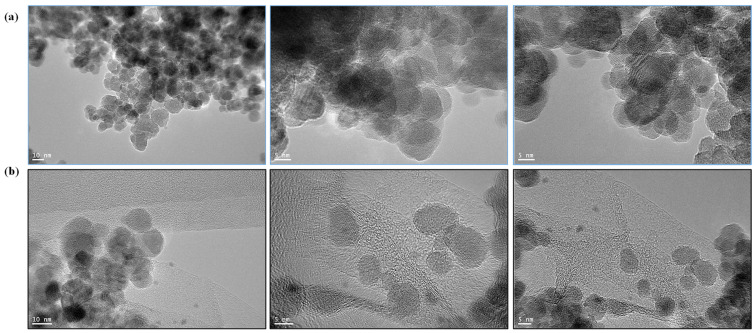
TEM of (**a**) Fe_3_O_4_ and (**b**) optimized Fe_3_O_4_/RGO/PEG nanocomposites.

The aqueous dispersions of Fe_3_O_4_ and its nanocomposites with RGO were also characterized using UV–Visible spectroscopy (Figure 3), where the characteristic peak for Fe_3_O_4_ was observed at ~390 nm [22], with minor red shifts in its peak noticed with the inclusion of RGO and PEG in the nanocomposites. The TEM images (Figure 4) further provided insights into the surface morphology and particle size of SPIONs as well as confirming the presence of RGO and PEG coating. Figure 4a presents the Fe_3_O_4_ QDs that were synthesized without the presence of GO. It is apparent that some level of agglomeration has taken place among these nanoparticles, which exhibit a predominantly spherical shape with a size ranging from 5 to 10 nm. These results align with findings from a previous study [23]. In Figure 4b, the TEM images of the Fe_3_O_4_/RGO/PEG nanocomposites are presented. At comparatively low magnification, Fe_3_O_4_ QDs with variable ellipsoid forms are evenly distributed and enveloped in RGO sheets. The geometric diversity could provide a larger surface area for their interactions with the tumor cells and potentially increase the efficacy of anticancer treatment when combined with targeted drug delivery.

### 3.2. Potential of Fe_3_O_4_ and Its Composites for Application in Magnetic Hyperthermia

**Figure 5:**
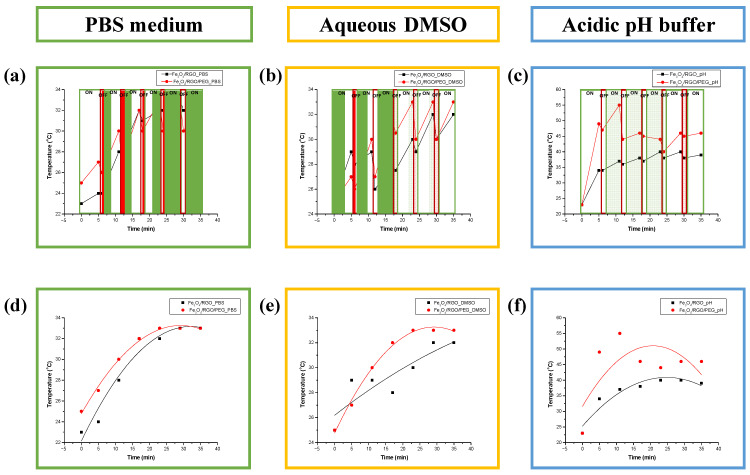
Heating profiles for solutions of Fe_3_O_4_/RGO (black) and Fe_3_O_4_/RGO/PEG (red) in (**a**,**d**) PBS, (**b**,**e**) aqueous DMSO and (**c**,**f**) acidic pH buffer upon exposure to magnetic field at 150 A.

The synthesized Fe_3_O_4_/RGO and Fe_3_O_4_/RGO/PEG nanocomposites were evaluated for their application in magnetic hyperthermia by exposing their dispersions to alternate magnetic fields in pulses over a period of 30 min, where various dispersions simulating the cell environments were investigated. The PBS and acidic pH buffer media were used to simulate the isotonic physiological environment often used in cell cultures and the acidic environment present around cancer cells [24], respectively. On the other hand, DMSO is a well-tolerated pharmaceutical agent that could minimize the aggregation of the hydrophobic nanoparticles in dispersions, though DMSO solutions above 1% concentration are toxic to cells. Nonetheless, the magnetic hyperthermia tests were conducted in 50% aqueous DMSO solutions to investigate the maximum achievable heating effects of the nanocomposites by excluding the influence of particle aggregation [25]. All aqueous solvents, without the nanocomposites being dispersed in them, did not show magnetically induced heating effects as also noted in our previous study [17]. The synthesized pristine Fe_3_O_4_ QDs considerably agglomerated during these investigations, irrespective of the dispersion media; thus, no appreciable heating effects were observed upon their exposure to the magnetic field. On the other hand, Fe_3_O_4_/RGO and Fe_3_O_4_/RGO/PEG exhibited considerable heating effects in various dispersion media upon exposure to the magnetic field, which was applied in pulses (5 min ON, 1 min OFF) over a total period of 35 min (Figure 5a–c).

The Fe_3_O_4_/RGO/PEG nanocomposites exhibited higher heating effects in all dispersion media (Figure 5) upon exposure to the magnetic field than the nanocomposites without PEG. However, the nanocomposites in the neutral PBS medium (Figure 5d) did not display any significant changes in the solution temperatures, with a gradual increase of 7 °C over a period of 35 min and a final temperature of ~32 °C. Similarly, the dispersions in aqueous DMSO solutions (Figure 5e) exhibited a gradual increase of 7–8 °C in the solution temperatures upon exposure to the magnetic field. Contrastingly, the dispersions in acidic pH buffer (Figure 5f) experienced a rapid change in solution temperature, with the temperatures reaching ~50 °C for Fe_3_O_4_/RGO/PEG nanocomposites within the first 5 min of exposure to the magnetic field. This heating effect achieved in the acidic media could be attributed to lower agglomeration of the potentially amphipathic nanocomposites due to their surface charges, which needs to be examined in future studies to elucidate this phenomenon [25]. Furthermore, the heating curves for all of the dispersions, irrespective of the inclusion of PEG, reached a plateau after 25 min of exposure to the magnetic field. This trend could be related to the phenomena governing heat generation by the magnetic nanoparticles, including associated changes in their relaxation energetics and the time required for magnetization reversal [26]. Additional studies on the magnetic and surface properties for these nanocomposites are needed to elucidate the predominant mechanisms governing heat generation under the magnetic field and to further optimize their dosage for practical applications while minimizing any inherent cytotoxic effects to the healthy cells.

### 3.3. Preliminary Investigation on Cytotoxic Effects of Fe_3_O_4_ and Its Composites

**Figure 6:**
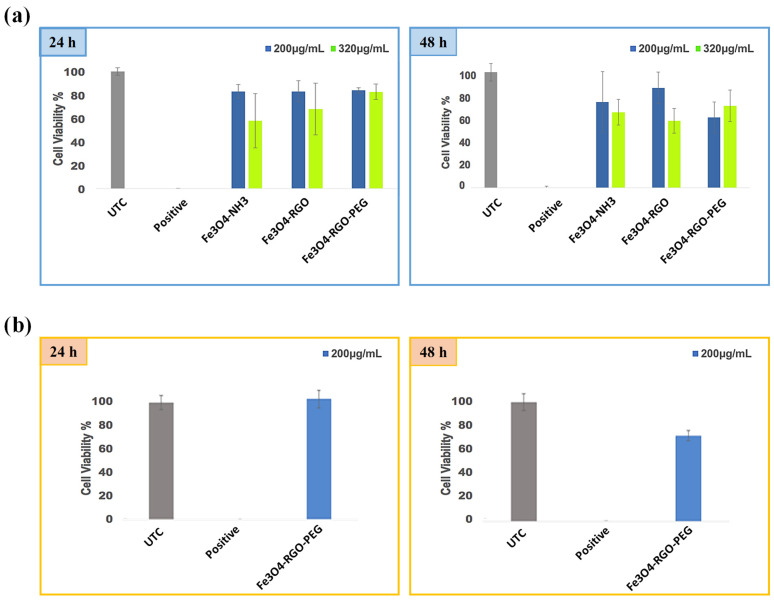
Cell viability of (**a**) CHO-K1 cells incubated with Fe_3_O_4_, Fe_3_O_4_/RGO and Fe_3_O_4_/RGO/PEG (200 and 320 µg/mL) over a period of 24 h and 48 h, and (**b**) HEK-293 cancer cells incubated with Fe_3_O_4_/RGO/PEG (200 µg/mL) at 24 and 48 h incubation time.

The synthesized pristine Fe_3_O_4_ and its nanocomposites with RGO exhibited minimal cytotoxic effects toward the healthy cells (CHO-K1) at a low concentration of 200 µg/mL over an incubation period of 24–48 h (Figure 6). Although these nanocomposites exhibited cytotoxic effects at the higher concentration of 320 µg /mL, the inclusion of PEG in the composites improved their biocompatibility for a longer incubation period (48 h). Despite the known biocompatibility of PEG, an anomaly was observed for Fe_3_O_4_/RGO/PEG at 200 µg/mL, which had lower cell viability than other samples; this may be attributed to unreacted reagents possibly onto these nanocomposites during the synthesis process [17]. Thus, future studies should employ additional purification steps for the synthesis of nanocomposites to eliminate this interference during cytotoxicity studies. Additional studies are also required to investigate the larger deviations observed for cell viabilities after 48 h incubation periods. Further studies are also required to analyze the inherent cytotoxicity of these samples on cancer cells in order to evaluate their efficacy systematically for in vitro anticancer therapy via magnetic hyperthermia.

## 4. Conclusions

To conclude, Fe_3_O_4_ QDs and their composites with RGO were synthesized via the coprecipitation method by fine-tuning the synthesis parameters, including reaction time, the concentration of ammonia and GO solutions and the inclusion of PEG for enhanced biocompatibility. These nanocomposites were evaluated in three dispersion media, including PBS, aqueous DMSO and acidic pH buffer, simulating typical cell environments for their potential application in magnetic hyperthermia. The dispersion of these nanocomposites in acidic pH buffer were observed to increase the solution temperature to 45 °C or higher within the first 5 min of exposure to the magnetic field as compared to other dispersion media, which highlighted their prospects for effective hyperthermia applications in the acidic environment present in the vicinity of cancer cells. Furthermore, these nanocomposites demonstrated low cytotoxic effects on healthy CHO-K1 cells. Extensive studies are required in future to investigate their potential cytotoxic effects on cancer cells as well as enhance their hyperthermic effects for in vitro and in vivo applications in targeted cancer therapies.

## Figures and Tables

**Table 1:** Average crystallite sizes for synthesized Fe_3_O_4_/RGO nanocomposites at given synthesis conditions.

Varied Synthesis Parameter	Synthesis Parameter Values	Fe_3_O_4_ Crystallite Size (nm)
Amount of NH_3_ added (mL) *	6	9.8
	8	13.0
	10	12.0
Reaction time (min) *	60	9.8
	75	11.0
	90	9.0
Amount of GO solution added (µL) for composites with RGO *	25 (0.1% GO)	11.0
	253 (1% GO)	12.6
	510 (2% GO)	14.0

* Fe_3_O_4_ QDs were synthesized under these reaction conditions without the inclusion of PEG.

## Data Availability

Experimental data will be available from the corresponding author on request.
